# Some Effective Factors on the Webometric Status of Selected Universities of Medical Sciences: Lessons Learned from Iran

**Published:** 2019-06

**Authors:** Nahid RAMEZANGHORBANI, Mehrnaz HAJIABEDIN RANGRAZ, Rosemarie NOOT HEIDARI

**Affiliations:** Department of Development and Cooperation of Information and Scientific Publications, Undersecretary for Research and Technology, Ministry of Health and Medical Education, Tehran, Iran

**Keywords:** Webometrics, Ranking, University, Iran

## Abstract

**Background:**

Web is considered an important tool for formal and informal communication and cooperation among members of the community including researchers. Universities’ websites played a significant role in the development of social structure through the creation of cyberspace. We aimed to evaluate the growth and Webometrics rankings of the country’s medical universities web sites base on interventional approach.

**Methods:**

This interventional study assessed Iranian universities of medical sciences’ websites from the periods of Jan 2015, Jul 2015 and Jan 2016. Medical universities websites were ranked based on four Webometrics indicators; PRESENCE, VISIBILITY, OPENNESS, and EXCELLENCE. To enhance the ranking of websites, 4 strategies were introduced in 3 seminars conducted from Jan to Jul 2015. Information needed from these 30 medical universities were obtained from Webometrics Ranking of World Universities based on 4 indicators and were collected in 2 steps (before and after introduction of strategies)

**Results:**

About 21% to 70% of the country’s medical universities have increased ranking after the interventions while 9% to 30% obtained a downward trend in their rankings, Tehran University of Medical Sciences obtained the highest rank followed by Shahid Beheshti University of Medical Sciences and the third rank was obtained by Shiraz University of Medical Sciences.

**Conclusion:**

The presence of websites play a vital role in the academic community. Doubtlessly, the idea of designing and launching a website without any knowledge of the principles and standards would be problematic and impossible.

## Introduction

The advent of the Internet has created radical changes not only in the collection- saving and sharing of information but also in revolutionizing mechanism needed to measure access to information. The current debate on online publications, the importance of access to information and the challenges posed by advocates of the traditional method have been taken more seriously. Currently, the Web is considered an important tool for formal and informal communication and cooperation among members of the community including researchers. The increasing number of indexed web pages has prompted the emergence of various methods of measurement and evaluation of web resources (1).

Universities’ websites have played a significant role in the development of social structure through the creation of cyberspace. These web-sites act as a multifaceted communication device and play significant role in introducing the scope and activities of the university e.g., research centers and hospitals affiliated to these universities, the duties, policies, colleges and departments, curriculum, degree programs, educational and research facilities, faculties, students and alumni. Presently, one of the most important factors in the success of academic institutions is the presence of a website and web access capabilities and most importantly the visibility of its website. One of the country’s priorities is to evaluate the academic institutions in terms of academic and research performance and the university’s website would serve as a tool in reaching this goal (2). The Shanghai Ranking System is considered one of the most outstanding academic ranking systems of the world, other important academic ranking systems include; the Times, QS Universities Rankings and HEEACT. Majority of these systems evaluate academic institutions based on the following indicators; academic achievements, quality of education, number of foreign students, number of Nobel Prize winners and scientific productions. Webometrics Ranking System evaluates the comprehensive overview of a university and academic institutions are ranked based on the following 4 indicators; number of web pages, the total number of PowerPoint, Word and PDF files available on the university’s website, the university’s qualitative articles must be among the 10% most cited articles in SCImago website and the number of external links that a website receives (3, 4).

However, in recent years, one influencing factors for the considerable growth of scientific productions is health sciences. Rankings of scientific output in the field of health sciences, supporting investments in research also serves as a guide to the development of scientific centers (5). Previous studies on website rankings in the domain of health draw significant results. About 56% of academic websites in terms of accessibility demonstrated unfavorable and very unfavorable condition and 60% has no link to their electronic publications and have no electronic resources on their websites (6).

Another study conducted on the trends of Webometric changes on Iranian Universities and Research Centers’ websites at national, regional and international levels has come to the conclusion that appropriate policies should be undertaken while meticulously monitoring academic websites by research policy makers and proper authorities in order to improve Webometric ranks (7, 8). One of the reasons for the medical universities’ low Webometric rankings is the lack of attention given by university authorities and the lack of necessary policies to improve website contents such as digital transformation of articles, dissertations and faculty members’ blogs (9, 10). Universities could contribute much in order to improve their Webometric rankings. Measures such as exhibiting their scientific achievements on their websites, providing access to electronic resources and important databases, free access to full-text resources and provision of the English language version and the inclusion of the hospitals’ and health care centers websites under the university’s domain are considered beneficial ( 4, 8, 10, 11). Moreover, the professional degree of faculty members in attracting links and sharing and the dissemination of bilingual information (local and English language) to users play an important factor in improving Web Impact Factor ranking (12, 13).

Considering the importance of Webometric Ranking as an important aspect of a quantitative study on the use of information resources, Structures and Web technologies and in portraying informatics and bibliometric approaches, the present study was conducted for the purpose of evaluating and improving the growth and Webometric rankings of the country’s medical universities web sites in order to attain a complete vision of the status of science and research in the domain of health at the national level and to attest the degree of their presence and success in this field.

## Methods

This interventional study assessed Iranian universities of medical sciences’ websites from the periods of Jan 2015, Jul 2015 and Jan 2016 respectively. Initially, by selecting the Asian continent and then selecting Iran, all medical universities were retrieved in terms of their rankings from the Webometrics database. Then the contents of each website were ranked based on Webometrics indicators (presented in webometrics info.) Webometrics Ranking was done at the national level and also trends of each university’s rankings were evaluated and analyzed throughout the study period. The growth trends of medical universities’ Webometric rankings were retrieved and results were compared using descriptive statistics (percentage and frequency, tables and graphs). Webometrics Ranking of World Universities is based on the analysis of 4 indicators; number of web pages, rich files such as the total number of PowerPoint, Word and PDF files available on the university’s website, the number of qualitative articles belonging to the 10% of highly cited articles in their respective fields in Scimago website ( https://www.scimagojr.com/) and the number of links and backlinks to the university’s website respectively. In order to enhance the ranking of websites, 4 solutions were introduced in the 3 seminars conducted from Jan to Jul 2015. Suggested strategies include:

- university’s publications should be under the university’s main domain,
- hospitals’ website, research centers, and health care centers in the urban and rural areas must be under the university’s main domain,
- Research centers affiliated with the university medical universities should refrain from having separate sites and instead use the university’s domain,
- Optimization of the site’s structure for search engines.

Moreover, in these conferences aside from providing solutions to improve accessibility and optimization of the website structure for search engines, exchanges of views, ideas, and academic experiences have been provided and also educational materials regarding the mentioned solutions were distributed in order to create necessary skills of the designated Webometrics staff in order that they will be able to make appropriate changes on their related websites.

## Results

Before implementing our intervention, medical universities rankings based on the Jan 2015 Webometrics Ranking results were assessed. Three of the country’s medical universities namely; Tehran, Shiraz, and Isfahan ranked first, second and third respectively. The rest of the country’s medical universities that ranked fourth to 30^th^ are written according to their ranks: Shahid Beheshti, Mashhad, Tabriz, Kerman, Ardebil, Zanjan, Kermanshah, Hamedan, Yazd, Arak, Mazandaran, Birjand, Iran, Golestan, Urmia, Kashan, Baqiyatallah, Kurdistan, Gilan, Qazvin, Social Welfare and Rehabilitation, AJA, Shahrekord, Babol, Bushehr, Jundishapur (Ahvaz), and Zahedan (Table 1).

**Table 1:** Rankings of the top 30 medical universities based Webometrics Rankings for the periods from (Jan and Jul 2015 and Jan 2016) respectively

***Row***	***Name of University of Medical Sciences***	***World Rank Jun 2016***	***World Rank July 2015***	***World Rank Jun 2015***	***Rate of Growth first 6 months***	***Rate of Growth second 6 months***	***Total Annual Growth Rate***
1.	*Tehran*	427	364	422	58	−63	−5
2.	*Shahid Beheshti*	901	924	1053	129	23	152
3.	*Shiraz*	1087	930	704	−226	−157	−383
4.	*Mashhad*	1286	1203	1151	−52	−83	−135
5.	*Isfahan*	1300	1184	981	−203	−116	−319
6.	*Tabriz and Health Services*	1350	1502	1458	−44	152	108
7.	*Kerman*	2072	2116	1665	−451	44	−407
8.	*Iran*	2114	2171	3173	1002	57	1059
9.	*Kurdistan Sanandaj*	2353	1735	3524	1789	−618	1171
10.	*Shahid Sadoughi*	2393	2378	2576	198	−15	183
11.	*Kermanshah*	2523	2568	2545	−23	45	22
12.	*Hamedan*	2533	2674	2558	−116	141	25
13.	*Mazandaran*	2547	2690	2694	4	143	147
14.	*Zanjan*	2582	2617	2493	−124	35	−89
15.	*Birjand*	2679	2576	2702	126	−103	23
16.	*Baqiyatallah*	2858	3030	3439	409	172	581
17.	*Guilan*	2863	2998	3599	601	135	736
18.	*Babol*	3031	3462	4849	1387	431	1818
19.	*Arak*	3049	3117	2616	−501	68	−433
20.	*Social Welfare and Rehabilitation Sciences Tehran*	3067	3311	3929	618	244	862
21.	*Kashan*	3185	3331	3429	98	146	244
22.	*Qazvin*	3205	3643	3698	55	438	493
23.	*Golestan*	3218	3375	3368	−7	157	150
24.	*Urmia*	3245	3282	3372	90	37	127
25.	*Bushehr*	3313	5661	4960	−701	2348	1647
26.	*Ardabil*	3412	3298	2406	−892	−114	−1006
27.	*Shahrekord*	3621	3917	4170	253	296	549
28.	*Zahedan*	3950	4228	6286	2058	278	2336
29.	Ahvaz Jundishapur	4800	5376	5430	54	576	630
30.	Aja	5916	5480	4160	−1320	−436	−1756

Following interventions, Webometrics ranking in Jul 2015 has shown the following results; Tehran ranking first with Shahid Beheshti ranking second and Shiraz on the 3^rd^ rank. The following 16 universities have shown improvements in their Webometrics Ranking; Zahedan, Kurdistan, Babol, Iran, Social Welfare and Rehabilitation, Gilan, Baqiyatallah, Shahrekord, Yazd, Shahid Beheshti, Birjand, Kashan, Urmia, Tehran, Qazvin, and Mazandaran respectively with Zahedan University of Medical Sciences obtaining a highly significant increase.

In the Jan 2016 Webometrics Ranking results, again the 3 medical universities Tehran, Shahid Beheshti and Shiraz obtained the highest results while the following 21 universities have shown improved rankings; Bushehr, Jundishapour (Ahvaz), Qazvin, Babol, Shahrekord, Zahedan, Social Welfare and Rehabilitation, Baqiyatallah, Golistan, Tabriz, Kashan, Mazandaran, Hamidan, Gilan, Arak, Iran, Kermanshah, Kerman, Urmia, Zanjan and Shahid Beheshti respectively while 9 medical universities namely Yazd, Tehran, Mashhad, Birjand, Ardebil, Isfahan, Shiraz, AJA and Kurdistan showed a downward trend in comparison to their 2015 Webometrics ranking results. Approximately 70% of these universities have improved their rankings while 30% have obtained a downward trend. Therefore, in the last year, 21%–72% of medical universities have increased their ranks while 9%–30% of these medical universities have obtained setbacks in their rankings.

**Fig. 1:**
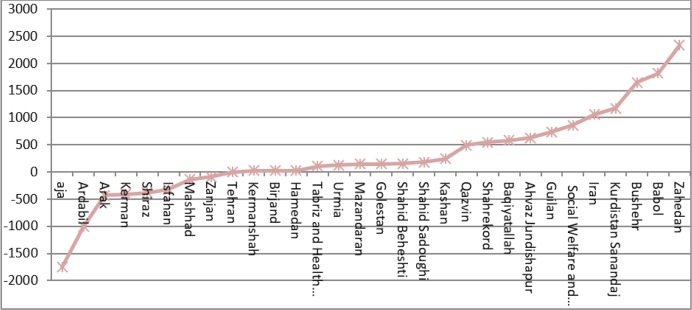
Medical Universities’ rate of improvement in Webometrics Ranking after the intervention

Webometrics Ranking after the intervention showed that Tehran University of Medical Sciences obtained the highest rank followed by Shahid Beheshti of Medical Sciences and the third rank was obtained by Shiraz University of Medical Sciences while Ahvaz Jundishapur University of Medical Sciences, Zahedan University of Medical Sciences and Shahrekord University of Medical Sciences have obtained the lowest ranks (Fig. 1).

## Discussion

Universities and research centers’ active presence on the web and the dissemination of findings in this manner is an indicator of their performance and also serves as a criterion for measuring development. Continuous evaluation of these web-sites is necessary in order to learn the level of their importance and to detect whether they have achieved their desired goals.

Assessment of the country’s universities and research centers’ websites basing on web-indicators has brought with it an advantage; the current situation and the performance of universities regarding the web environment has caught the attention of policymakers and on the other hand, heads and managers of these organizations are trying their best to provide the necessary requirements for their university and research center in accordance to international standards in order to stay actively in the web environment and gain a better reputation and credibility so, in this direction, the present study aimed at evaluating the county’s medical universities Webometrics’ Ranking has been conducted.

In the 2016 Webometrics Ranking results 21% to 70% of the country’s medical universities have increased ranking after the interventions while 9% to 30% have obtained a downward trend in their rankings and based on this result, Tehran University of Medical Sciences obtained the highest rank followed by Shahid Beheshti University of Medical Sciences and the third rank was obtained by Shiraz University of Medical Sciences while universities that obtained the lowest rank after the intervention include; Ahvaz Jundishapur University of Medical Sciences, Zahedan University of Medical Sciences and Shahrekord University of Medical Sciences respectively. Of course one of the reasons for this negative fluctuation could be attributed to the changes in the method of calculating the Excellence indicator while other reasons could be attributed to the effect of sanctions that the country has been facing in recent years but the most important reason is the lack of high visibility and university’s website being considered to be the core of its academic existence. For universities to attain high visibility of their websites the following items must be considered; number of pages, language, rapid update and systematic information, rich content, varied and useful information, simple navigation, global scope, electronic journal(s) and full text articles so that links from other websites and provision of Union Catalog and online access public catalog will be received. This result is consistent with the result of Danesh et.al. Administrators and designers of websites under study should plan and identify factors that attract links in a website in order to improve the quality and contents of their respective websites. However, the ultimate success of a website depends on factors such as quality, size, language, duration of website and other related factors and not be limited to one or two factors to be considered as the only reasons for the success of a website (14, 15).

The 2015 World University Rankings Report published by the Scientific Information Database (SID), in this particular period of time the number of universities having obtained a rank of 500 and higher have maintained a stable rank while for universities that obtained the rank of 1000, only 3 universities have indicated an increase in ranking (16). With little effort, both Shiraz University of Medical Sciences and Shahid Beheshti of Medical Sciences will probably be among the top ranked 1000 universities (2016 Scientometrics Center). In this period of time some universities namely; Zahedan, Babol, Iran, Kurdistan and Shiraz showed a growth rate of 25%.

The results of the 2015 Leiden Ranking based on bibliographic data from the Web of Science database has included 13 Iranian universities among the rank of the world’s top 750 universities having the highest number of scientific publications. In the 2013 ranking results only 5 Iranian Universities were included in the list and in 2014 an additional 7 universities were included in the rank and in the 2015 and 2016 rankings with the inclusion of Shahid Beheshti University of Medical Sciences and Isfahan University of Medical Sciences the total number of universities included in Leiden Ranking has reached to 14 (17). Moreover, in the study conducted on 15 universities’ websites has stated that in terms of influence Iranian academic websites have attracted lesser audience due to the language barrier. Middle Eastern websites are a mixture of different languages such as Persian, Arabic, Kurdish, Turkish and Hebrew. Therefore, the implementation of bilingual website on every medical university is of outmost importance which is one of the proposed solutions mentioned in this study. Some of the medical universities websites are providing the English and Persian languages to their audiences (18). In study “Government portal effectiveness: managerial considerations for design and development” was attempted to develop a comprehensive framework for website management taking into considerations the strict compliance of the website’s design and computer programming (19).

Moreover, basing on this study, management considerations played a vital role in web design. Discussion regarding the design of a university’s website is one of the important things that should be taken into account. Absence of academic websites would create confusion and deprive the academic society of scientific information. The process of SID in this frenzied business information can be useful to the country’s academic society (6).

By closely examining research, the presence of such websites could play a vital role in the academic community. Doubtlessly, the idea of designing and launching such website without any knowledge of the principles and standards would be problematic and impossible. On the other hand, in considering the sensitivity of the faculty members in obtaining the desired scientific information within the least possible time can give us the conclusion that provision of a well-organized website in terms of visual effects and systematic search strategies and yet constant is an undeniable principle which can significantly alter the flow of information to users in a positive direction. Moreover, an improvement in the ranking of university’s websites based on the criteria of increased ranking on different search engines particularly the Google Search Engine can then be realized.

The limitation of this study is in many cases; some universities have changed this web domain, but keeping older ones or even organized with two domains. These actions caused to decrease the visibility and web rank.

## Conclusion

In considering the evaluation results of websites being studied, universities concerned should exert more efforts in improving the status of their websites in terms of content and design. Implementing appropriate measures such as provision of personalized pages for faculty members and graduate students in order that they can be actively involved in content production, provision of an English version of their website and or increasing their website’s number of pages, accessibility on updated educational, scientific and academic resources and news in the English language, embed informative and educational files and course content with due consideration to the author’s rights in the form of rich files and the publication of scientific e-journals through their respective website. Careful observance of these measures would lead to an increase in the number of content resulting in increased web-pages, impact and visibility and, consequently, increases the chances of a greater connection between users and search engines. Additionally, website designers and managers should strictly observe the website optimization standards such as the use of hypertext markup language tags and the use of keywords related to the website content.

## Ethical considerations

Ethical issues (Including plagiarism, informed consent, misconduct, data fabrication and/or falsification, double publication and/or submission, redundancy, etc.) have been completely observed by the authors.
